# Magnitude and causes of first-line antiretroviral therapy regimen changes among HIV patients in Ethiopia: a systematic review and meta-analysis

**DOI:** 10.1186/s40360-019-0361-3

**Published:** 2019-11-01

**Authors:** Zerihun Ataro, Birhanu Motbaynor, Fitsum Weldegebreal, Mekonnen Sisay, Tewodros Tesfa, Habtamu Mitiku, Dadi Marami, Zelalem Teklemariam, Zewdneh Shewamene

**Affiliations:** 1 Department of Medical Laboratory Sciences, College of Health and Medical Sciences, Haramaya University, P.O.Box, 235 Harar, Ethiopia; 2 Department of Pharmaceutical Chemistry, School of Pharmacy, College of Health and Medical Sciences, Haramaya University, P.O. Box, 235 Harar, Ethiopia; 3 Department of Pharmacology and Toxicology, School of Pharmacy, College of Health and Medical Sciences, Haramaya University, P.O.Box, 235 Harar, Ethiopia; 4 Western Sydney University, Sydney, Australia

## Abstract

**Background:**

Antiretroviral therapy (ART) has markedly decreased the morbidity and mortality due to HIV/AIDS. ART regimen change is a major challenge for the sustainability of human immunodeficiency virus (HIV) treatment program. This is found to be a major concern among HIV/AIDS patients in a resource-limited setting, where treatment options are limited.

**Objectives:**

The aim of this review is to generate the best available evidence regarding the magnitude of first-line antiretroviral therapy regimen change and the causes for regimen change among HIV patients on ART in Ethiopia.

**Methods:**

The reviewed studies were accessed through electronic web-based search strategy from PubMed Medline, EMBASE, Hinari, Springer link and Google Scholar. Data were extracted using Microsoft Excel and exported to Stata software version 13 for analyses. The overall pooled estimation of outcomes was calculated using a random-effect model of DerSimonian–Laird method at 95% confidence level. Heterogeneity of studies was determined using I^2^ statistics. For the magnitude of regimen change, the presence of publication bias was evaluated using the Begg’s and Egger’s tests. The protocol of this systematic review and meta-analysis was registered in the Prospero database with reference number ID: CRD42018099742. The published methodology is available from: https://www.crd.york.ac.uk/PROSPERO/display_record.php?RecordID=99742.

**Results:**

A total of 22 studies published between the years 2012 and 2018 were included. Out of 22 articles, 14 articles reported the magnitude of regimen change and consisted of 13,668 HIV patients. The estimated national pooled magnitude of regimen change was 37% (95% CI: 34, 44%; Range: 15.1–63.8%) with degree of heterogeneity (I^2^), 98.7%; *p*-value < 0.001. Seventeen articles were used to identify the causes for first-line antiretroviral therapy regimen change. The major causes identified were toxicity, 58% (95% CI: 46, 69%; Range: 14.4–88.5%); TB co-morbidity, 12% (95% CI: 8, 16%; Range: 0.8–31.7%); treatment failure, 7% (95% CI: 5, 9%; Range: 0.4–24.4%); and pregnancy, 5% (95% CI: 4, 7%; Range: 0.6–11.9%).

**Conclusions:**

The original first-line regimen was changed in one-third of HIV patients on ART in Ethiopia. Toxicity of the drugs, TB co-morbidity, treatment failure, and pregnancy were the main causes for the change of the first-line regimen among HIV patients on antiretroviral therapy.

## Background

Worldwide, a total of 36.7 million individuals were infected with human immunodeficiency virus (HIV) [1]. In June 2017, 53% of all people living with HIV were receiving antiretroviral therapy (ART) [2]. Sub-Sahara Africa remains the region most heavily affected by HIV. In eastern and southern Africa, a total of 19 million individuals were living with HIV [1] and 11.7 million individuals received antiretroviral treatment, representing 60% of HIV positive individuals in the region in 2016 [2]. Similarly, HIV infection in Ethiopia continues to pose a threat to the lives of people. Based on a report from Ethiopian demographic health survey (EDHS), the estimated prevalence of HIV was 1.2% among men and women of the 15–49 age group in Ethiopia [3]. The number of people enrolled in ART rose from 900 in 2005 to 300,000 in 2010, and 436,000 in 2017 and the number of facilities providing ART services increased from four in 2003 to 481 in 2009 and 1361 in 2017 [4–6].

Highly active antiretroviral therapy (HAART) changes HIV/AIDS from diseases with a high mortality rate to manageable chronic diseases by decreasing the progression of AIDS and reducing HIV-related illness and deaths. Treatment change and poor adherence, however, restrict the therapeutic success and sustainability of the original regimen. In addition to the poor adherence to the antiretroviral drug, severe adverse effects of the drug and treatment failure complicate the entire management of antiretroviral therapy [7–9]. HAART regimens commonly require changes, which often involve switches of multiple medications simultaneously. As a result, treatment change of therapy has become a common phenomenon and hence limitation of treatment option has turned out the major concern of the future HAART and HIV/AIDS patients [10, 11].

Ineffectiveness of antiretroviral therapy and regimen change can be caused by many factors. A rationale for therapy change can be acute and chronic toxicities, concurrent clinical situations, development of treatment failure, poor adherence, a sub-optimal regimen, co-morbidity and a pregnancy desire [12, 13]. Change in the regimen lead in a number of challenges; reducing both the duration and the possibility of viral control due to cross-resistance between different alternative drugs and overlapping toxicity between and within an antiretroviral drug class. Subsequently, the likelihood that successful HAART will last a life time is poor [14–16]. Therefore, optimizing the limited available combined anti-retroviral regimen is essential in order to improve the long term access and sustainability of HIV treatment program.

Unless absolutely needed, ART regimen is not changed. Once a drug combination is modified, it can no longer be given to the same patient again because it causes significant morbidity and poor quality of life [17]. Most ART programs in low- and middle- income settings follow the World Health Organization (WHO) ART guidelines that emphasize a public health approach to ART delivery [18]. This strategy aims at maximizing population-level survival through a standardized sequencing of available antiretroviral drugs, delivered to individuals by means of simplified approaches to clinical decision making and basic laboratory monitoring [19].

Changing of ART regimen is based on clinical parameters and the drugs are chosen on the basis of their demonstrated efficacy in suppressing HIV replication and improving survival of PLHA, low cost and wide availability [20]. However, concerns have emerged about the durability, safety, tolerability and rational use of ART regimens. Non-rational use of ART regimen affects the primary goal of ART, i.e. suppression of the viral load; restoration and preservation of immunologic function and improved quality of life may not be achieved**.** It is important to study the long term outcome of the ART regimen used and design strategies that increase the durability of the original regimen.

Even though a public health approach ART of WHO guideline has been used in developing countries [18], there is a difference in the implementation of the guideline of ART regimen change, adherence of the ART treatment across different countries. Furthermore, the national guidelines may differ in their choice of the regimens based on the economic status and capacity [21, 22]. This systematic review included studies conducted in Ethiopia with the aim of providing local evidence as per the real setting of the population. This assists the clinicians to focus on the most effective treatment combinations.

In a resource-limited setting like Ethiopia, where treatment options are limited, it is essential to design strategies to increase the durability of the original regimen. So as to achieve this goal, it is important to determine the magnitude and causes of initial HAART regimen change. However, except primary studies conducted in different part of Ethiopia, the magnitude of change of first-line ART regimen and the causes responsible for the change are not well studied in a systematic review approach. Therefore, this systematic review is aimed to determine the rate of the initial ART regimen change and its causes among HIV patients on ART.

The growing access to ART in Ethiopia brings a complex set of issues; when to initiate therapy, what regimen to use, what drugs to use within each class, when to change therapy, and what alternative drugs to use. Data on change of highly active antiretroviral therapy provide scientific information for clinicians, policy and decision-makers on the long term strategic approach to initial and subsequent decisions regarding ART. It assists the clinicians to focus on the most effective treatment combinations. Furthermore, it helps to design appropriate measures to increase the duration of the original regimen among patients on antiretroviral therapy which subsequently preserve the future treatment options.

## Methods

### Study protocol

The Preferred Reporting Items for Systematic Reviews and Meta-analysis (PRISMA) guideline was used to report the finding of this review [23]. This systematic review and meta-analysis was conducted using the PRISMA checklist. The protocol of this systematic review and meta-analysis was registered in the Prospero database with reference number ID: CRD42018099742. The published methodology is available from: https://www.crd.york.ac.uk/PROSPERO/display_record.php?RecordID=99742

### Identification of records and search strategy

The search strategy aims at finding studies that are both published and unpublished studies. We searched the following electronic databases: Medline, EMBASE, Hinari, Springer link and Google Scholar. A three-step search strategy was utilized in this review. An initial restricted search of the digital databases was undertaken, followed by an assessment of the text phrases contained in the title and abstract, and the index terms used to define the article. A second search was then performed across all included databases using all recognized keywords and index terms. Thirdly, additional studies have been searched for the reference list of all identified articles. Subject headings relevant to each database were used, for example, MeSH for Medline. In addition, google and hand searching was employed to retrieve grey literature**.** Initial keywords used were: ART, antiretroviral, HAART, chang*, Shift*, Switch*, modif*, substitut*, Ethiopia. The retrieval was limited to English Language.

### Study selection

Records obtained from various digital databases were exported to Endnote reference software version 7 (Thomson Reuters, Stamford, CT, USA). We used Endnote reference manager to remove duplicated studies. In order to identify studies that might fulfill the inclusion criteria, two reviewers (ZA and BM) independently screened the titles and abstracts. Studies that are deemed to meet inclusion by title/abstract screening undergone full-text appraisal by two authors (ZA and BM) independently for methodological validity using standardized critical appraisal instruments from the Joanna Briggs Institute Meta-Analysis of Statistics Assessment and Review Instrument (JBI-MAStARI). The remaining authors played an important role in resolving discrepancies between two authors in order to reach a consensus. Any disagreements between the reviewers were solved by discussion. Full texts of included articles were extracted systematically by using a standardized data extraction tool from the Joanna Briggs Institute Meta-Analysis of Statistics Assessment and Review Instruments.

### Inclusion and exclusion criteria

During the screening and assessing of full texts for eligibility, there were predefined inclusion-exclusion criteria to arrive at the final included papers. Observational studies such as prospective and retrospective cohort studies, and analytical cross-sectional studies conducted in Ethiopia addressing the magnitude and/or cause of first-line ART regimen change were included. Studies published in the English language were included. All review articles and original articles conducted outside of Ethiopia were excluded during initial screening. Articles with outcome measures that were unrelated, missing or insufficient and articles with irretrievable full texts were excluded.

### Data extraction

With the help of standardized data abstraction format prepared in Microsoft Excel, two authors (ZA and BM) independently extracted necessary data from the articles. Information about the primary author, study setting, study design, age, region, year of study, year of publication, number of study participants, time of data collection, number of cases (first-line ART regimen change), and number of each causes reported for regimen change were extracted from each study.

### Critical appraisal of studies

To maintain methodological validity, before inclusion of the articles two independent reviewers assessed the articles using the Joanna Briggs Institute (JBI) critical appraisal checklist for studies reporting prevalence data [24]. The assessment tool consisted of nine questions about the quality of the study for which articles receive values representing the extent to which they met the following criteria: Yes, No, Unclear and Not applicable. This critical appraisal was conducted to assess the internal (systematic error) and external (generalizability) validity of studies and to reduce the risk of biases. The mean score of the two authors was taken for final decision and studies with a score greater than or equal to five out of nine were included.

### Outcome measurements

The primary outcome measure is the magnitude of first-line ART regimen change in Ethiopia. The number of each case (i.e. first-line regimen change due to any reason) from each article was weighed based on its sample size, pooled and measured using percentage. The review’s secondary outcome is the causes of the first-line ART regimen change. The purpose of this review is to assess the national pooled estimates of first-line ART regimen change and the causes for the first-line ART regimen change among HIV patients.

### Data processing and statistical analysis

A Microsoft Excel prepared format was used to extract data from the included studies. The extracted data were exported to Stata software version 13 for analyses. Considering the variation in true effect sizes across the population, the random effects model of Der Simonian and Laird was applied at 95% confidence level for the analyses. Heterogeneity of studies was determined using I^2^ statistics. For the magnitude of regimen change, Begg’s and Egger’s tests were used to evaluate the presence of publication bias [25, 26]. Funnel plots of log odds versus 1/SE (log odds) were constructed for the outcome. A statistical test with a *p*-value less than 0.05 was considered significant.

## Results

### Search results

**Fig. 1:**
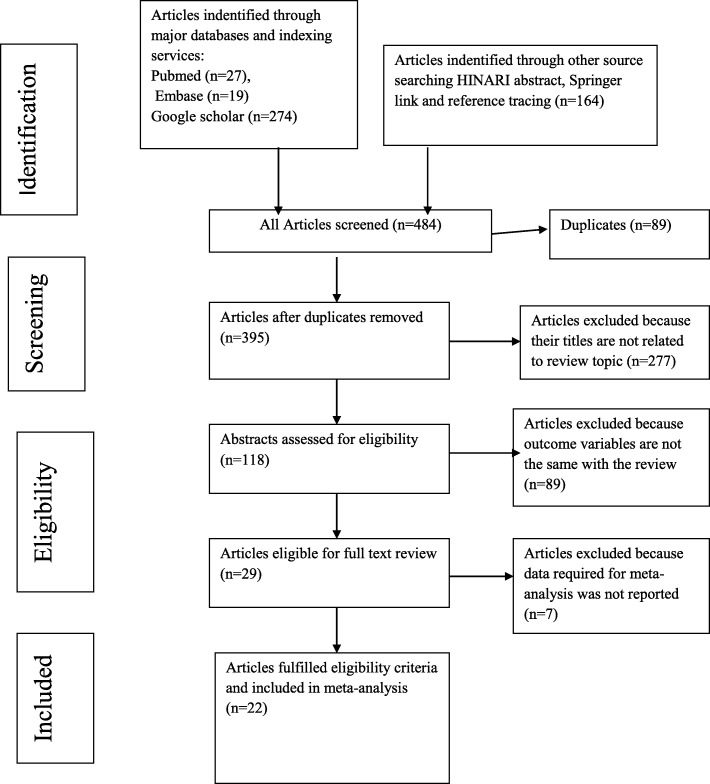
PRISMA flow chart describing the selection process

The systematic review and meta-analysis was performed according to the PRISMA statement [23]. In our literature search, a total of 484 records were identified from several sources, including PubMed/Medline, EMBASE, Google Scholar, HINARI and Springer link. Using Endnote and manual tracing, 89 duplicate articles were removed. A total of 395 records were screened using their titles and 277 of them were excluded. The remaining 118 records were screened using their abstracts and 89 of them were excluded. Full texts of 29 articles were then evaluated for eligibility. From these, the outcome of interest of 7 articles was found missing and/or insufficient and they were excluded. Finally, 22 articles that passed the eligibility criteria and quality assessment were included in this study (Fig. 1). These articles were divided into two groups: those which reported the magnitude of regimen change, and those which reported the causes for regimen change. Some studies reported both of them.

### Study characteristics

A total of 22 studies were included in this systematic review and meta-analysis (Table 1). Included studies were published between the years 2012 and 2018. We included studies that employed both retrospective and prospective cross-sectional study design. All of the included studies were not carried out in the same age group. Four studies were done on children [27–30] and thirteen studies were done on adult participants [15, 31–42]. Four studies had included all the age ranges [43–46].

**Table 1:** Characteristics of studies describing the magnitude and causes of first-line regimen change

Author (year of publication)	Quality Score	Study design	Study setting (region)	Year of study	Included Age group (in year)	Sample size	Number of cases (first line ART regimen change)	Number of each causes/reasons reported for regimen change	No	%
Wube M et al. (2013) [31]	7	Retrospective cross sectional	Nekemt hospital,	2010	≥15 years	142	NA	Toxicity/side effect	114	80.3
								Pregnancy	9	6.3
								TB co morbidity	8	5.6
								Drug stock out	7	4.9
								Treatment failure	4	2.8
Zeleke A (2016) [27]	6.5	Retrospective cross sectional	University of Gondar Hospital	2014	< 15 years	225	101 (44.9%)	Side effects	29	12.9
								Diagnosis of TB	32	14.2
								Program shift from D4T to AZT or TDF	32	14.2
								Stock out of drugs	5	2.2
Sisay MS et al., (2018) [28]	7	Retrospective follow up	Selected hospitals in Amhara National Regional State	2016	< 15 years	824	299 (36.3%)	Drug stock out	145	48.5
								Drug side effects/toxicity	72	24.1
								Treatment failure	67	22.4
								New TB case	8	2.7
								Other	7	2.3
Jima Y et al. (2013) [15]	7	Retrospective cross sectional	Selected health facilities in Addis Ababa	2010	≥18 years	300	NA	Toxicity	195	65.0
								TB Co-morbidity	75	25.0
								Pregnancy	15	5.0
								Treatment failure	9	3.0
								Adherence difficulty	6	2.0
Anlay DZ et al. (2016) [32]	8	Retrospective follow up	University of Gondar Hospital	2015	≥15 years	410	88 (21.5%)	Side effects	62	70.45
								Tuberculosis	18	20.45
								Pregnancy	4	4.5
								Virological failure	3	3.4
								Occurrence of hepatitis B with chronic liver diseases	1	1.1
Kasu B et al. (2017) [43]	6.5	Retrospective cross sectional	Asella referral Hospital	2015	All	1468	221 (15.1%)	Drug toxicity	84	38.0
								Treatment failure	54	24.4
								Poor adherence	51	23.1
								Pregnancy	21	9.5
								Co-morbidity due to TB	11	4.9
Gebremedhin L et al. (2014) [44]	6	Retrospective cross sectional	Ayder Referral Hospital, Mekelle	2013	All	720	157 (21.8%)	Toxicity	119	75.8
								Tuberculosis	22	14.0
								Treatment failure	15	9.6
								Pregnancy	1	0.6
Teklay G et al. (2013) [45]	7.5	Retrospective cross sectional	Jimma University Specialized Hospital	2012	All	403	257 (62.8%)	Weight gain	129	50.2
								Toxicity	77	30.0
								TB treatment interaction	44	17.1
								Pregnancy	8	3.3
								Treatment failure	1	0.4
Geremew A et al. (2014) [33]	7.5	Retrospective cross sectional	Jimma University Specialized Hospital	2013	≥ 18 years	324	121 (37.3%)	Toxicity/side effect	86	71.1
								Treatment failure	25	20.7
								Pregnancy	7	5.8
								New TB	3	2.5
Mulugeta A et al. (2012) [34]	7	Cross sectional	Dessie Regional Referral Hospital	2007	≥ 18 years	122	NA	Toxicity	80	65.6
								TB Co-morbidity	17	13.9
								Pregnancy	14	11.5
								Treatment failure	8	6.6
								Adherence difficulty	3	2.4
Woldemedhin B et al. (2012) [46]	6	Retrospective cross sectional	Hawasa referral Hospital and shashemene referral Hospital	2010	All	340	NA	Toxicity	230	67.6
								Co-morbidity with TB	65	19.1
								Pregnancy	36	10.6
								Treatment failure	9	2.6
Assefa D et al. (2014) [35]	6.5	Retrospective cross sectional	Fitche Hospital	2013	≥ 18 years	68	NA	Toxicity	56	72.7
								Co-morbidity TB	2	2.6
								New drug available	7	9.1
								Treatment failure	11	14.2
								Patient refused to take the drug	1	1.3
Yirdaw B et al. (2014) [42]	6	Retrospective	Felege Hiwot referral hospital	2013	≥ 15 years	387	189 (49.2%)	NA		
Kassie Y et al. (2014) [36]	6	Retrospective cross sectional	Bedele Hospital	2013	≥ 18 years	84	NA	Toxicity	63	75.0
								Co-morbidity due to TB	10	11.9
								Pregnancy	10	11.9
								Treatment failure	1	1.2
Haile D, et al. (2016) [37]	7	Retrospective cohort study	Selected Hospitals in Bale	2014	≥ 15 years	4809	1630 (33.9%)	NA		
Tadesse W et al. (2014) [38]	7.5	Cross sectional	University of Gondar Hospital	2012	≥ 18 years	384	78 (20.3%)	Adverse drug reaction	62	79.5
								Treatment failure	10	12.8
								Tuberculosis co-morbidity	4	5.1
								Pregnancy	2	2.6
Bilie B et al. (2017) [39]	7.5	Retrospective	Jimma University Specialized Hospital	2013	≥ 18 years	1284	615 (47.9%)	Toxicity	301	48.9
								TB Comorbidity	88	14.3
								Phaseout	124	20.2
								Pregnancy	24	3.9
								Treatment failure	4	0.6
								Drug out of stock	3	0.5
								Hepatitis	1	0.2
								Unknown	70	11.4
Tadesse B et al. (2017) [29]	8	Prospective cohort	Selected hospitals in Southern Nations Nationalities and Peoples Region	2016	< 18 years	628	253 (42.6%)	Toxicity or side effects	39	14.7
								TB co-infection	2	0.8
								National Guideline Change	187	70.3
Yassin S et al. (2017) [30]	7	Retrospective cohort	Fiche and Kuyuhospitals	2015	< 15 years	269	131 (48.7%)	NA		
Bokore A, et al. (2018) [40]	7	Retrospective cross sectional	East and west Wollega health institutions	2017	≥ 18 years	243	NA	Peripheral neuropathy	146	60.1
								Hepatotoxicity	22	9.1
								d4t faith out	18	7.4
								CNS toxicity	16	6.6
								Anemia	16	6.6
								Rash	15	5.3
								Others	10	4.9
Mekonnen E et al. (2018) [41]	7.5	Retrospective cohort	Jimma University Tertiary Hospital	2014	≥ 18 years	1533	731 (47.7%)	Drug toxicities	431	58.9
								A new TB treatment	120	16.4
								Pregnancy	29	3.9
								Treatment failure	24	3.3
								Others	127	17.4

*Abbreviations*: *NA* Not applicable, *TB* Tuberculosis, *CNS* Central Nervous System, *AZT* Zidovudine, *TDF* Tenofovir, *d4T* Stavudine

### Magnitude of drug regimen changes

**Fig. 2:**
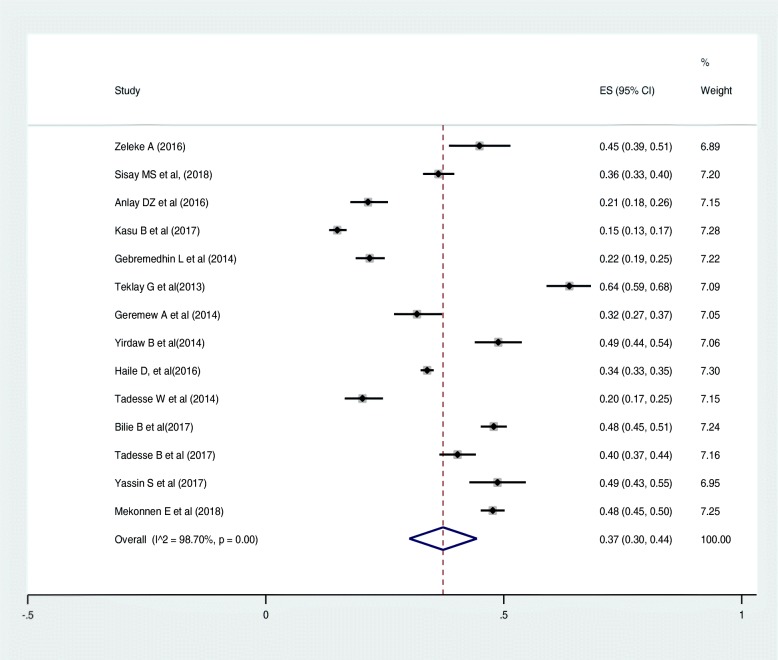
Pooled estimate of magnitude of regimen change in Ethiopia

Out of the 22 articles included in this review, 14 articles reported the magnitude of regimen change, varying from 15% [43] to 64% [45]. The sample size ranges from 225 [27] to 4809 [37] patients. A total of 13,668 patients were included in all the studies. The estimated national pooled magnitude of regimen change was 37% (95% CI: 34, 44%) with degree of heterogeneity (I^2^), 98.7%; *p*-value < 0.001 (Fig. 2).

### Causes of regimen change

Out of the 22 articles included in this review, 17 articles reported the causes for first-line ART regimen change. Out of the 17 articles, 12 articles mentioned drug toxicity, as a cause for a regimen change [15, 29, 31, 33, 34, 36, 39, 41, 43–46], three articles described it as a side effect [27, 28, 32], one article describes the reason as adverse drug reaction (ADR) [38]. In this systematic review, the causes described as a side effect, hepatotoxicity and ADR were categorized under toxicity. One study described the causes (with their numbers) as hepatotoxicity, peripheral neuropathy, central nervous system (CNS) toxicity, anemia, and rash separately [40]. We grouped all this under toxicity.

**Fig. 3:**
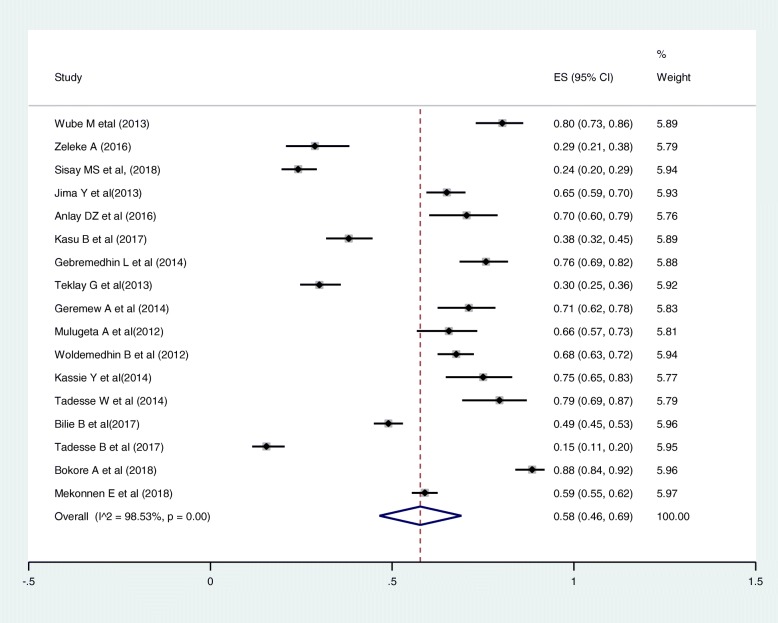
Pooled estimates of magnitude of toxicity as a cause for first line regimen change

**Fig. 4:**
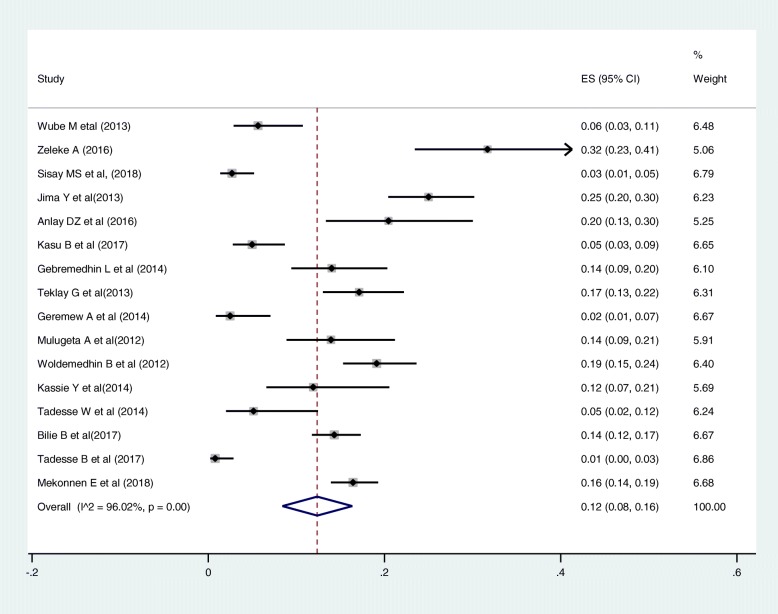
Pooled estimates of the magnitude of TB co-morbidity as a cause for first line regimen change

**Fig. 5:**
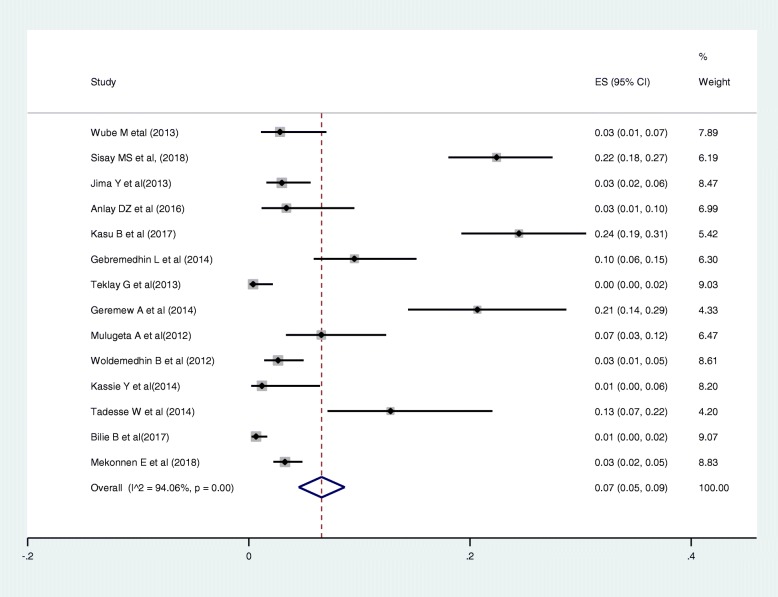
Pooled estimates of the magnitude of treatment failure as a cause for first line regimen change

**Fig. 6:**
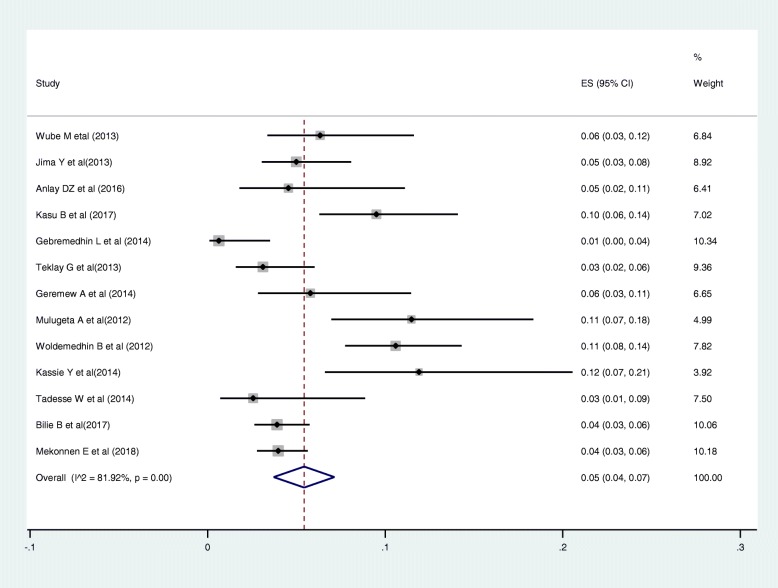
Pooled estimates of magnitude of pregnancy as a cause for first line regimen change

Out of the 17 articles included in the assessment of the causes of magnitude change, 13 articles described pregnancy [15, 31–34, 36, 38, 39, 41, 43–46]. Fourteen articles described treatment failure [15, 28, 31–34, 36, 38, 39, 41, 43–46], two studies mentioned clinical failure [28], and virological failure [32] as a cause. Out of the 17 articles included in the assessment of the causes of magnitude change, only one article didn’t mention TB co-morbidity as a cause [40]. The most commonly mentioned causes for first-line ART regimen were toxicity, 58% (95% CI: 46, 69%; Range: 14.4–88.5%) (Fig. 3); TB co-morbidity, 12% (95% CI: 8, 16%; Range: 0.8–31.7%) (Fig. 4); treatment failure, 7% (95% CI: 5, 9%; Range: 0.4–24.4%) (Fig. 5); and pregnancy, 5% (95% CI: 4, 7%; Range: 0.6–11.9%) (Fig. 6).

The other reasons responsible for the first-line ART regimen change were stock out problem, 14% (95% CI: 0, 29%) and guideline change, 33% (95% CI: 7, 60%). Out of the 17 articles, stock out problem was reported by 4 articles [27, 28, 31, 39] and national guideline change was reported by four articles and it was due to phasing out of stavudine from the NRTI backbone [27, 29, 39, 40].

### Publication bias

**Fig. 7:**
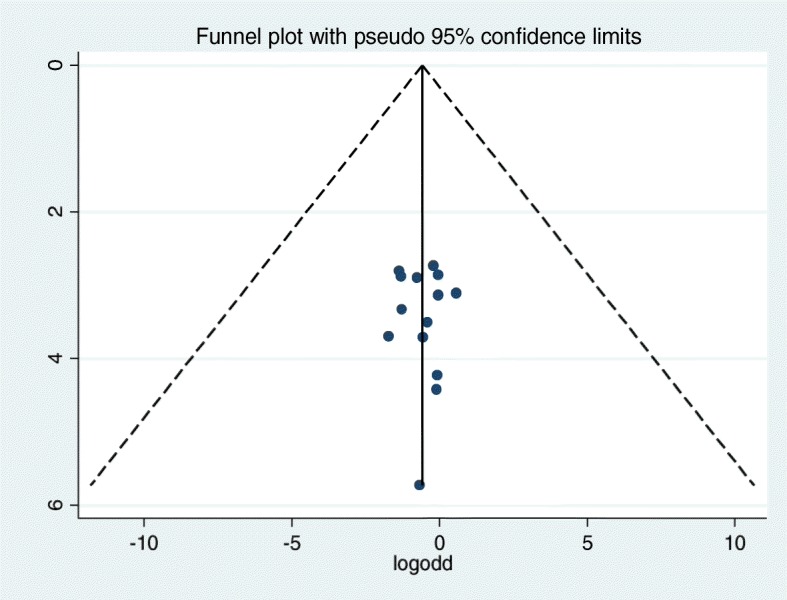
Funnel plot depicting publication bias

Funnel plots of log odds versus 1/SE (log odds) were constructed for the outcome (magnitude of regimen change) and the statistical test confirmed that there is no evidence of publication bias reporting the magnitude of regimen change (Egger’s test, *p* = 0.915; Begg’s test, *p* = 0.956) (Fig. 7).

## Discussion

This systematic review and meta-analysis was conducted to estimate the national pooled magnitude of first-line regimen change among HIV patients in Ethiopia**.** A total of 22 original studies addressing the magnitude of regimen change and/or the causes for regimen change among HIV patients in Ethiopia (in resource-poor settings) within the specified timeframe were included.

This review revealed that 37% of HIV patients were changed their first-line regimen. The regimen change mentioned consists of any regimen change, i.e. those recommended by the physicians as well as those due to different causes. Regardless of the cause, a high rate of first-line regimen change was reported in this systematic review. This may indicate poor adherence by patients and/or poor compliance to guidelines by prescribers. Treatment success needs strict lifelong drug adherence and strong guideline compliance in order to achieve potentially lifelong suppression of HIV replication.

The result of this meta-analysis was lower to a cohort study conducted in Europe and North America, which reported 40.3% of patients modified first-line ART [47]. In Swiss HIV cohort studies, higher rates of change were reported in first line ART regimen [10]. In a study that compared the approaches to ART showed that, in South Africa, 22% patients subsequently changed their first-line regimens from more than 2000 patients receiving HAART in two towns in Cape Town, South Africa. While in Switzerland, 53% of patients subsequently changed their first-line regimens out of 1000 patients enrolled in the Swiss [48]. The difference in the rate of regimen change might be due to the difference in socio-demographic characteristics, healthcare systems, variation in defining regimen change and study population. The other possible reasons might be availability viral load testing for monitoring of treatment response might pick regimen change due to virological failure earlier in some countries. Furthermore, limited options of antiretroviral regimen may limit the clinician decision on first-line ART regimen change.

Upon initiation of antiretroviral therapy, patients usually stay permanently on medications. Antiretroviral therapy’s main objectives are, to keep peak viral load suppression, restore and preserve immunologic function, improve quality of life, and decrease morbidity and mortality associated with HIV. This is achieved through properly regulated antiretroviral therapy [49]. However, there are many factors that lead to change/modification of HAART combination and switch of HAART treatment. These include long term toxicity and/or adverse drug reaction, treatment failure (including virological, immunological and clinical failure), poor adherence, a desire for pregnancy, and/or co-morbidity [12, 50–53]. This regimen change affects the success of the treatments to achieve the goal of the United Nations Program on HIV and AIDS (UNAIDS) [54].

Based on the available data, this systematic review and meta-analysis revealed to synthesize the evidence on potential causes of first-line regimen change in Ethiopia. It indicated that the toxicity (58%), tuberculosis (TB) co-morbidity (12%), treatment failure (7%) and pregnancy (5%) were the top four causes for the change of the first-line regimen. Other causes indicated were program shift and stock out of the drug. Even though the causes for first-line ART regimen change are in line with the recommendation of ART treatment outcomes, the rate of ART regimen change was found to be high. This provides important information for clinicians and policymakers in the country in order to evaluate and plan for the future needs of the ART treatment program.

Toxicity was found to be one of the major causes for the first-line ART regimen change in this review. This finding is consistent with several primary studies done in different countries [12, 51, 55, 56]. In all of these primary studies, the most predictable cause for ART switching was the toxicity of the drugs. The proposed reason for this is primarily an inherent property of the drugs that trigger changes to the ART regimen. Furthermore, it is due to the fact that the occurrence of side effect alters the quality of life and even result in the death of a patient and it is a short-term predictor for regimen change. The most commonly encountered toxicity related causes for regimen changes ranges from simple up to life-threatening adverse effects. These include nausea, rash, anemia and peripheral neuropathy, hepatotoxicity, mitochondrial damage, and bone marrow toxicity [57].

Toxicity is a potential threat to the effectiveness of ART. ART regimen change may be an alternative for the toxicity management, but it should be done considering the risk of loss of future therapy choices. In health institutions, less toxic and better-tolerated treatment choices for HIV should be accessed and used more frequently. The majority of patients included in the studies used for this systematic review were started stavudine based regimens [29, 34, 36, 39, 41, 44, 46]. Stavudine is known by its high toxicity which is found to be the major reasons for the removal of stavudine from the current regimen and currently new patients with HIV/AIDS are not starting with stavudine containing regimens. Furthermore, the occurrence of high rate toxicities could be attributed to advanced HIV infection. The majority of the patients included in the articles of this review had advanced disease stage at baseline (indicated by WHO clinical stage III and IV) [39, 41, 44, 46]. Patients with advanced disease stage were more likely to change the initial HAART regimen. Drug toxicity adversely affects the quality of life and potential for optimum adherence. Lack of adherence ultimately leads to the emergence of resistance to antiretroviral drugs and treatment failure.

TB-HIV co-morbidity was the second major reason for changing the first-line ART regimen in this review. This might be attributed to the infectiousness properties of the TB bacilli that cause an opportunistic infection at any level CD4 count in HIV infected patients. The occurrence of TB-HIV co-morbidity after 6 months of initiation of ART predicts clinical failure that indicates the need for treatment change [58].

There was a higher chance of changing a first-line ART regimen in patients who did receive other medications other than ART. TB drugs especially rifampicin induces cytochrome 450 enzymes that facilitate the metabolic activity of the liver, which makes under the therapeutic level of ART drugs especially nevirapine [59]. Nevirapine containing regimens were responsible for TB related switches due to the drug-drug interaction that exists between nevirapine and rifampicin. In order to avoid this drug interaction, clinicians replace the nevirapine with efavirenz based regimens that have lesser interaction with rifampicin [19, 59].The other explanation might be the pill burden which is to be taken simultaneously to treat both AIDS and opportunistic infection resulting in poor adherence and its common toxicity with ART drugs result in the need for change [60].

Treatment failure is another factor for treatment modification. Treatment failure is described as disease progression after initiation of ART and occurs when the anti-HIV treatments can’t control the infection. Antiretroviral treatment failure is associated with virologic failure, immunologic failure, and/or clinical failure [61]. The monitoring of first-line treatment failure and the decision to start second-line ART regimen are largely based on the clinical and immunologic assessment of patients. In a resource-limited setting like Ethiopia, viral load monitoring, which is the gold standard technique for diagnosing ART failure, is not widely available.

Clinico-immunologic criterion leads to the unnecessary changing of first-line ART regimen to second-line ART regimen in the absence of virologic failure and a late change to second-line ART regimen. This late change of first-line ART regimen is related to accumulation of mutation and results in resistance to other drugs that might could be used as a second-line alternative [62, 63]. Optimal management of HIV-infected patients receiving ART using the viral load test is important for early detection of treatment failure. Strong HIV treatment program in the monitoring of patients on first-line treatment to identify those who are more likely to develop treatment failure is highly crucial.

Planning pregnancy or being pregnant was also the reason for changing first-line ART regimen in this review. In Ethiopian, ART treatment change for efavirenz-based regimens is recommended during pregnancy [64]. This shift was primarily due to the teratogenic effect of efavirenz, which should be removed during the first-trimester of the pregnancy. However, another study showed that efavirenz has similar safety during pregnancy like the other ART’s and had no increased risk of overall birth defects [65].

### Strength and limitation

This is the first systematic review and meta-analysis conducted in Ethiopia to show the national pooled estimates of the magnitude of first-line ART regimen change among HIV infected patients. This review adhered to established standard methods for conducting systematic reviews. Furthermore, the absence of publication bias found in this meta-analysis increases the certainty of this evidence on decision making and resource utilization for possible preventive measures.

Interpretations of the current study findings should consider the following important limitations. The analysis included papers with various definitions of regimen change. The reviewed articles have also difference in study design, the type of statistical methods, and the variables included in the analysis. These variations may have resulted in selection bias or low statistical power, thus hindering results. Exploring the sources of heterogeneity was limited by an insufficient number of the included studies. Only a few studies were carried out with the same age group, which created some difficulties in conducting sub group analysis (pooling of age-specific prevalence)**.** Similarly, we were unable to carry out a sub group analysis using different characteristics. In addition, the analysis was limited to only articles published in the English language; which may have precluded relevant articles.

## Conclusion

This systematic review showed that the original first-line regimen was changed in one-third of HIV patients on ART in Ethiopia, which was found to be high. The proportions of individuals who changed the first-line ART regimen in our resource-constrained country present a challenge to the our limited treatment choices. Toxicity of drugs, TB-HIV co-morbidity, treatment failure, and pregnancy were the main causes for the change of the first-line regimen. Designing strategies to increase the durability of the original regimen are essential in such resource-limited setting, where treatment options are limited. Selection of the right antiretroviral regimen and appropriate care, close follow-up and frequent laboratory result monitoring after ART initiation is necessary to maintain patients on their initial regimen.

## Data Availability

All the data is contained within the manuscript.

## Abbreviations

ADR: Adverse drug reaction
ART: Antiretroviral therapy
HAART: Highly active antiretroviral therapy
HIV: Human immunodeficiency virus
PRISMA: Preferred Reporting Items for Systematic Reviews and Meta-analysis (PRISMA)
TB: Tuberculosis
